# Synergistic Interactions within Disturbed Habitats between Temperature, Relative Humidity and UVB Radiation on Egg Survival in a Diadromous Fish

**DOI:** 10.1371/journal.pone.0024318

**Published:** 2011-09-08

**Authors:** Michael J. H. Hickford, David R. Schiel

**Affiliations:** Marine Ecology Research Group, School of Biological Sciences, University of Canterbury, Christchurch, New Zealand; National Institute of Water & Atmospheric Research, New Zealand

## Abstract

Anthropogenic impacts, including urbanization, deforestation, farming, and livestock grazing have altered riparian margins worldwide. One effect of changes to riparian vegetation is that the ground-level light, temperature, and humidity environment has also been altered. *Galaxias maculatus*, one of the most widely distributed fishes of the southern hemisphere, lays eggs almost exclusively beneath riparian vegetation in tidally influenced reaches of rivers. We hypothesized that the survival of these eggs is greatly affected by the micro-environment afforded by vegetation, particularly relating to temperature, humidity and UVB radiation. We experimentally reduced riparian vegetation height and altered shading characteristics, tracked egg survival, and used small ground-level temperature, humidity and UVB sensors to relate survival to ground-level effects around egg masses. The ground-level physical environment was markedly different from the surrounding ambient conditions. Tall dense riparian vegetation modified ambient conditions to produce a buffered temperature regime with constant high relative humidity, generally above 90%, and negligible UVB radiation at ground-level. Where vegetation height was reduced, frequent high temperatures, low humidity, and high UVB irradiances reduced egg survival by up to 95%. Temperature effects on egg survival were probably indirect, through reduced humidity, because developing eggs are known to survive in a wide range of temperatures. In this study, it was remarkable how such small variations in relatively small sites could have such a large effect on egg survival. It appears that modifications to riparian vegetation and the associated changes in the physical conditions of egg laying sites are major mechanisms affecting egg survival. The impacts associated with vegetational changes through human-induced disturbances are complex yet potentially devastating. These effects are particularly important because they affect a very small portion of habitat that is required to complete the life history of a species, despite the wide distribution of adults and juveniles across aquatic and marine environments.

## Introduction

Alterations to the riparian zone on coastal plains have occurred worldwide as urbanization, coastal development and flood control measures increase [1]. The impacts producing change include channelization of streams and rivers, increased water flow and scouring, deposition of sediments from catchments, changes to vegetation, and alteration of the light, temperature and humidity environment. Taken together, these have resulted in often disastrous consequences to the structure of habitats and the species that rely on them for all or part of their life histories. This is particularly so for diadromous fishes during the transition between freshwater and marine habitats. Numerous impacts on populations have been well-documented, such as for salmon, that require transit pathways up rivers and high-quality areas for spawning redds, both of which have been compromised in many areas [2]. Lesser known, but equally important in a cultural and fisheries context, are impacts to smaller diadromous fishes such as *Galaxias maculatus* (Jenyns, 1842), one of the most widely distributed species of the southern hemisphere [3].

*G. maculatus* forms the basis of a culturally important fishery in New Zealand in which juveniles (‘whitebait’) are netted as they migrate back into freshwater from their marine larval stages [4]. Although there are five species of whitebait in New Zealand, around 95% of the whitebait returning from offshore is *G. maculatus* [5], [6]. Statistics on this fishery are not kept, but there is considerable anecdotal evidence that catches have declined in the past few decades [7]. The degree to which overfishing is responsible for this decline is debatable, but there is no doubt that there has been extensive destruction or alteration of the habitats required for egg-laying and development [8].

The success of *G. maculatus* spawning and egg development is closely associated with the composition and physical characteristics of their riparian spawning habitat [9]. *G. maculatus* spawns during autumn in upper river estuaries, immediately after new or full moon spring tides when the tidal range is maximal [10], [11]. Eggs are laid and fertilized in riparian vegetation that is inundated only by the highest spring tides [12]. An initial adhesive coating on the newly deposited eggs sticks them to the stems and aerial root-mass of riparian vegetation [11] where they develop supratidally for c. 28 days. Egg hatching occurs within minutes of inundation on the next set of spring tides [3]. Tidal and riverine currents then sweep the newly hatched larvae (5–7 mm) out to sea as the tide falls. After a 4–6 month pelagic development period [13], post-larval juveniles return to rivers by sensing freshwater plumes following rain events [4].

*G. maculatus* eggs are laid preferentially beneath particular species of grasses, which leads to increased survival compared to eggs laid in the other types of vegetation present [9]. Although predation of eggs by exotic slugs and mice has been reported [14], [15], it is highly variable spatially and temporally [16], and of far less importance than abiotic factors in determining egg survival [17]. The physical characteristics of the riparian vegetation, however, are strongly associated with egg laying and survival, especially the height and density of the vegetation which greatly influence ground-level temperature and humidity. These qualities of the riparian vegetation should also influence the light environment, especially the exposure of developing *G. maculatus* eggs to ultraviolet (UV) radiation.

Increased levels of the UVB portion (280–320 nm) of solar radiation are known to cause adverse effects on many species, particularly their early life stages (see [18] for review). For example, fish embryos in several species had greater mortality when exposed to higher levels of UVB [19], [20], [21], [22]. There is a suggestion, supported by preliminary work, that phototoxicity, particularly to UVB, may negatively affect *G. maculatus* egg survival. The LD_50_ for *G. maculatus* eggs was found to be only 3.6 kJm^−2^ (Hickey C.W., unpublished results), which equates to less than one hour of exposure to clear summer UVB levels in New Zealand [18]. Because New Zealand is naturally exposed to high levels of UVB radiation [23] and human-induced reductions in stratospheric ozone have allowed more solar UVB radiation to reach the earth's surface in recent decades [24], it may well be the case that there are impacts on *G. maculatus* egg survival, especially as spawning habitats are altered. Furthermore, anthropogenic stressors, particularly bank alteration and livestock grazing in the extensive rural areas of New Zealand, have severely compromised riparian vegetation in areas where much of the whitebait production occurs [9].

We tested the hypothesis that the quality of riparian vegetation around micro-sites of egg masses affects survival of *G. maculatus* eggs, specifically with respect to temperature, relative humidity, and UVB radiation. We experimentally reduced the height of riparian vegetation, tracked egg survival and used small ground-level sensors to relate egg survival and the abiotic effects of altered vegetation.

## Materials and Methods

### Ethics Statement

The research presented in this manuscript complied with the requirements of the University of Canterbury's Animal Ethics Committee Code of Ethical Conduct. The experimental study was done on private land. All necessary permissions and permits were obtained from the landowners before observational and field studies were initiated.

### Experimental protocol

Because *G. maculatus* eggs are laid and develop over one lunar month, the habitat characteristics during this short interval are most relevant to egg survival. Consequently, we selected plots where recently laid eggs were in high densities and used these for experimental manipulations. All experimental plots were within 5×2.5 m exclosures established in 2007 which prevented access to livestock and allowed regeneration of riparian vegetation. These were located at Goughs Bay, Banks Peninsula, New Zealand (−43.8073° E, 173.0906° S; see [9] for details of exclosures). On 29 March 2011, immediately after a spring tide spawning episode, eight exclosures were searched for *G. maculatus* eggs and 18 masses containing at least 10 eggs per cm^2^ were marked. Fertilized eggs are only 1.2 mm in diameter, so a mass of 100 cm^2^ contained at least 1000 eggs. These were then randomly allocated to treatments within a fully orthogonal experiment to test the factors of vegetation height (3 levels) and light (2 levels), with three replicates of each. Small data loggers immediately adjacent to egg masses were used to gauge physical variables in each treatment combination and egg survival was the response variable.

To gauge control conditions, the species composition and height of vegetation within a 500×500 mm area surrounding each egg mass were measured. The initial vegetation height across all plots was 303±24.9 mm (mean ± SE). Six of the 18 egg masses were then allocated to each height treatment of ‘Uncut’ (no manipulation), ‘½ Cut’ resulting in a vegetation height of 146.7±61.7 mm, and ‘Cut’ leaving a vegetation height of 61.7±2.1 mm. The manipulations were done with grass shears, being careful not to disturb root-masses and eggs. Once the vegetation had been trimmed, eggs were counted in a marked quadrat (100×100 mm) placed haphazardly inside the egg mass and the density of vegetation was estimated by counting the number of stems in three 50×10 mm transects across the quadrat. Half of the egg masses within each vegetation height treatment were then randomly allocated either to ‘Shaded’ or ‘Open’ treatments. The shaded treatment reduced the direct light and incident solar UVB radiation reaching the vegetation and eggs beneath. It consisted of a 500×500 mm canopy of monofilament shade cloth (Shaded - Premium knitted HDPE, shade factor 80%, Agpac Ltd) fitted to a thin wire frame that kept it 100 mm above the top of the vegetation. With a nominal Ultraviolet Protection Factor of 5, it should have allowed only 20% of UVB radiation to pass through it without impeding the flow of air or water. Across the treatments, the ‘Uncut Open’ replicates had no manipulations and acted as controls.

To determine environmental correlates around the eggs within each treatment, ground-level temperature and relative humidity were measured every 2 minutes with Hobo Pro v2 temperature/RH loggers (Onset Computer Corp) that were placed adjacent to the marked quadrat. UVB radiation reaching the egg masses was measured every 5 seconds using UV dosimeter badges (31 mm ø×12 mm; Scienterra Ltd) that were placed at ground level adjacent to the marked quadrat. Ambient environmental variables were measured by attaching loggers to a fence post 800 mm above ground level.

To determine egg survival in a single cohort, eggs in all treatments were recounted prior to hatching and instantaneous survival rates were calculated. At the same time, vegetation heights and densities were remeasured.

### Data analysis

Differences in the growth and density of vegetation at the beginning and end of the experiment were tested with a General Linear Model (GLM). All temperature and humidity data were used in analyses, but UVB irradiance data between the hours of 1900 and 0700 (darkness) were discarded. A one-hour moving average was applied to all temperature, relative humidity and UVB irradiance data to remove the influences of short-term fluctuations. Frequency (%) distributions of temperature, relative humidity and UVB irradiance were generated for all treatments.

Egg densities across the experimental plots were tested with a GLM to determine if there were any initial differences. These were not significant but, because there were slight initial differences among treatments, we used initial egg density as a covariate in an analysis of egg survival across treatments at the end of the experiment.

Tukey's HSD post hoc tests were used when GLM indicated significant effects. A Bonferroni correction was applied when multiple significance tests were used. When necessary, egg survival data were arcsine transformed prior to GLM to satisfy the assumption of homogeneity of variance as detected by Cochran's tests [25].

To test the association between egg survival and the physical environment, maximum (temperature and UVB irradiance) and minimum (relative humidity) values from the frequency distributions of the Uncut (Control) plots were used as breakpoints for regression analysis. The amount of time (%) each treatment spent beyond these breakpoints was included as a covariate.

To test how much of the variability in *G. maculatus* egg survival was accounted for by the biological and physical (predictor) variables, and to find the subset of the variables that best explained variability in the survival data, a non-parametric multivariate regression analysis was done using the DISTLM module in PERMANOVA [26], [27]. Initially, individual predictor variables were analyzed separately (ignoring other variables) in marginal tests for potential relationships with the egg survival data. Variables were then subjected to stepwise forward-selection procedure (sequential test, R^2^ selection criteria), where the amount of variability explained by each variable added to the model is conditional on the variables already in the models. P-values for the marginal tests were obtained using 9999 permutations of the normalized predictor data, while conditional tests were done using 9999 permutations of residuals under the reduced model. All tests were based on Euclidean dissimilarities, calculated among arcsine transformed survival observations. Principal Coordinates analysis (PCoA) was used to visualize the relationships between egg survival data and the biological and physical predictor variables. All multivariate analyses were done using PRIMER 6 and PERMANOVA+ [28].

## Results

### Riparian Vegetation

The most common components of riparian vegetation were exotic pasture grasses: *Agrostis stolonifera* (84.8±4.7% cover), *Schedonorus phoenix* (11.4±4.4%) and *Holcus lanatus* (2.5±0.3%). After trimming, vegetation density averaged 3.6±0.2 stems cm^−2^ across the experimental plots. During the two week experiment, the vegetation in the trimmed treatments grew at a greater rate than that in the Uncut plots (F_2,12_ = 102.89, p<0.001, Tukey HSD, p<0.001), and the vegetation in the Open plots grew at a greater rate than that in the Shaded plots (F_1,12_ = 23.29, p<0.001). By the end of the experiment, the grasses in the Uncut, ½ Cut and Cut plots had grown 2.5±1.1, 22.0±3.7 and 32.8±2.3 mm respectively, and there was a slight increase in stem density in the Cut plots (by 0.3±0.1 stems cm^−2^, F_2,12_ = 67.583, p<0.001). Even with these changes, however, there were still noticeable differences among treatments at the end of the experiment.

### Physical Environment

**Figure 1:**
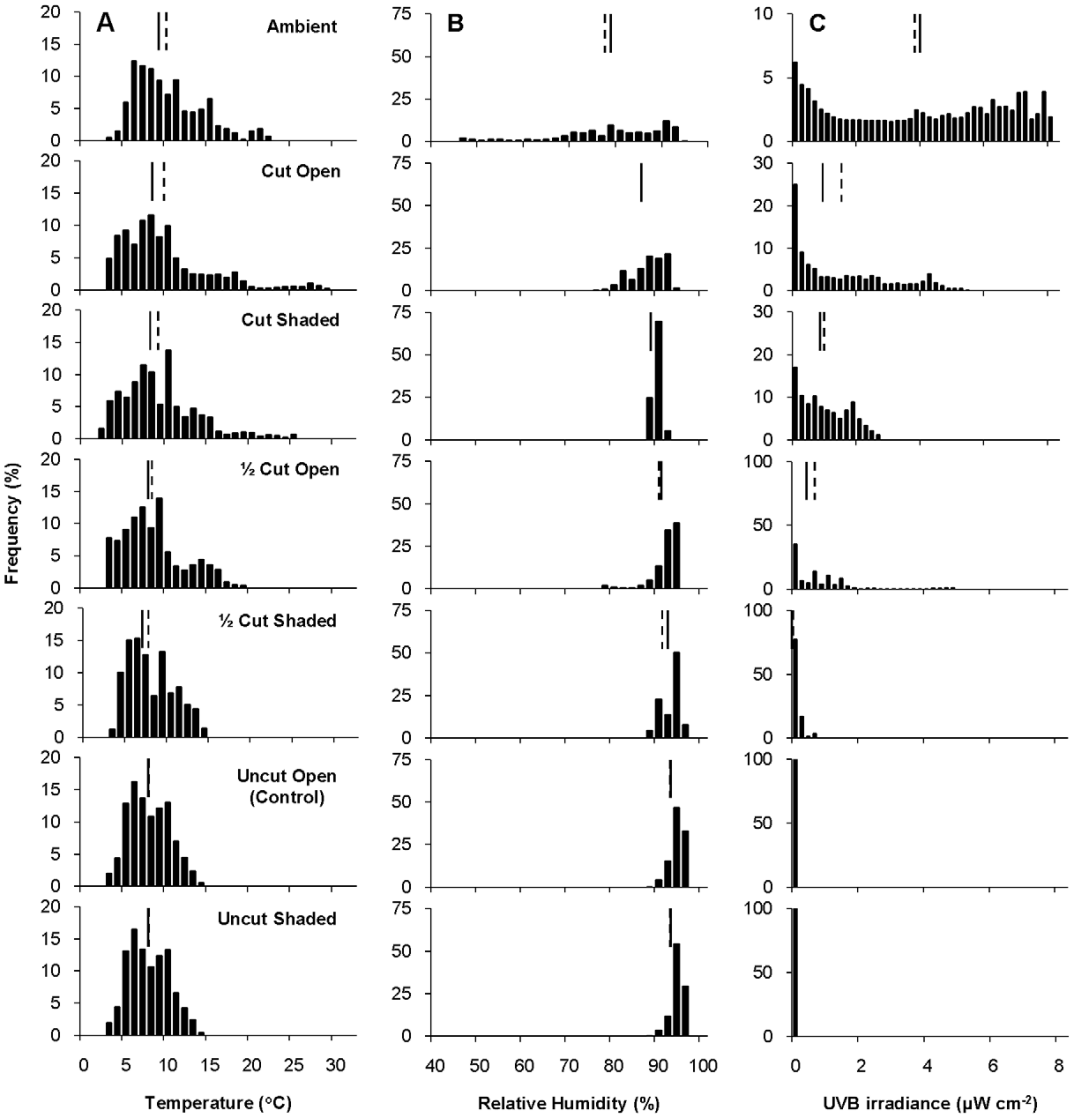
Frequency (%) distributions of environmental variables across experimental treatments. (A) temperature, (B) relative humidity, and (C) UVB irradiances at ground-level in experimental plots and in ambient conditions. Note differing y-axis scales on the UVB irradiance panels. Median (solid line) and mean (dashed) are shown on each panel.

The ground-level physical environment in all of the experimental plots was markedly different from the surrounding ambient conditions, and there was a gradient of temperature reduction from ambient across the experimental vegetation heights (Fig. 1). The shifting median temperature across experimental treatments was a reflection of the higher temperatures being truncated as grass height increased, indicating that there was a buffering effect of grasses on ground-level temperature. For example, the median ground-level temperature of 8.1°C in the Uncut Open (i.e., Control) plots was 1.6°C lower than the ambient air temperature and 0.6°C lower than that in the Cut Open plots (Fig. 1a). More importantly, however, the Control plots experienced a maximum temperature of 15°C in Shaded and Open treatments, whereas the ½ Cut treatment temperatures exceeded 20°C and Cut treatments reached 30°C. Using the maximum temperature of 15°C from the Control as a reference, the ground-level temperature in all ½ Cut and Cut treatments (Shaded and Open) exceeded 15°C for 12.1% of the time on average. However, the temperature in the Uncut plots exceeded 15°C for only 0.5% of the time. The shading treatment had a minimal effect on temperature by reducing the median ground-level temperature of all trimmed treatments by 0.2°C on average.

Relative humidity levels were also greatly different between ambient and ground-level, and among experimental treatments (Fig. 1b). The increasing height of grasses increased the average humidity around the egg masses below by truncating the less humid ambient conditions. The median relative humidity in the Uncut Open (Control) plots was 93.5%, which was 13.4% higher than the median ambient relative humidity and 6.6% higher than that in the Cut Open plots. Ground-level relative humidity in all ½ Cut and Cut treatments (Shaded and Open) fell below 90% (the minimum humidity of the Control) for 23.3% of the time on average. Relative humidity in the Uncut plots fell below 90% for only 0.4% of the time. The shading treatment increased the median relative humidity of all ½ Cut and Cut treatments by just 1%.

Of all the physical variables, the level of UVB irradiance had the greatest response to experimental treatments (Fig. 1c). The frequency distribution of ambient UVB irradiances was relatively uniform during the experiment, but peaked at over 8 µW cm^−2^. The Cut Open plots were closest to ambient in UVB irradiance levels and were very different from the Uncut and ½ Cut treatments. Shading reduced UVB irradiance in the Cut and ½ Cut plots, but both still encountered considerable periods (83.0% and 22.3% respectively) above 0 µW cm^−2^. The UVB irradiance in the Uncut plots was always around 0.

**Figure 2:**
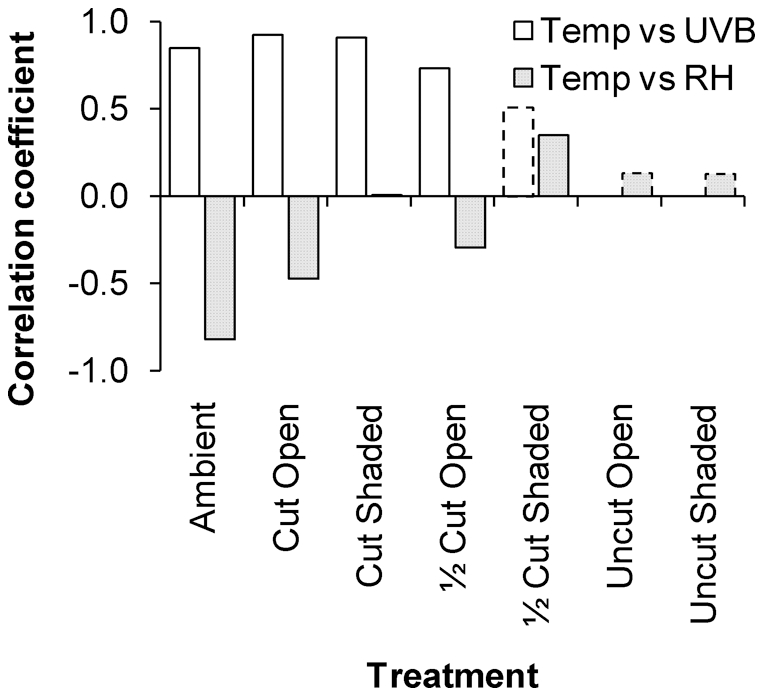
Correlations between environmental variables across experimental treatments. Pearson's correlation between average hourly temperature, UVB irradiance (n = 22) and relative humidity (n = 163) at ground-level in experimental plots and in ambient conditions. Bars with dashed borders represent correlation coefficients that were not significant.

Over all the experimental treatments, the Cut Open plots had the highest temperatures, lowest humidity and highest UVB irradiance levels, followed by the ½ Cut Open and Cut Shaded treatments. However, there were significant correlations between the levels of the physical variables, the strength of which decreased with shading and vegetation height (Fig. 2). Average hourly ambient temperature was closely associated with UVB irradiance (r_20_ = 0.847, p<0.001) and relative humidity (r_161_ = −0.819, p<0.001). The positive association between temperature and UVB irradiance in the trimmed Open plots and the Cut Shaded plots was absent in the ½ Cut Shaded and Uncut plots. The negative association between temperature and relative humidity in the trimmed Open plots was absent in the trimmed Shaded and Uncut plots. As more riparian vegetation was removed, the relationship between temperature, relative humidity and UVB irradiance became more similar to that of the ambient conditions. Tall, dense riparian vegetation modified ambient conditions to produce a buffered temperature regime with constant high relative humidity and negligible UVB irradiance at ground-level.

### Egg survival

**Figure 3:**
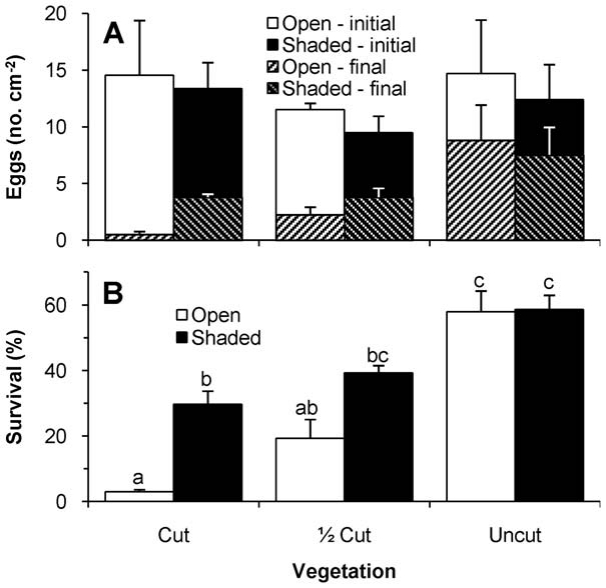
*Galaxias maculatus* egg densities and survival across experimental treatments. Mean (+ SE) (A) initial and final densities and (B) survival of *Galaxias maculatus* eggs in Open and Shaded treatments of three vegetation trimming treatments. Equivalent letters above the bars in (B) indicate statistically homogeneous groups as determined by Tukey HSD with p<0.05.

Initial egg densities averaged 12.7 cm^−2^ across all experimental plots (Fig. 3a). Although variable, there were no significant differences in initial densities among the vegetation height (F_2,12_ = 0.678, n.s.) or shading treatments (F_1,12_ = 0.488, n.s.). Egg survival ranged from c. 58% in the Shaded and Open Uncut plots to 3.0±0.7% in the Open Cut plots (Fig. 3b). Egg survival did not depend on initial densities (F_1,11_ = 1.013, n.s.). There was a significant interaction between the vegetation height and shading treatments on egg survival (F_2,11_ = 4.767, p<0.05). In the ½ Cut and Cut plots, survival was lower in the Open plots than in the Shaded plots.

**Figure 4:**
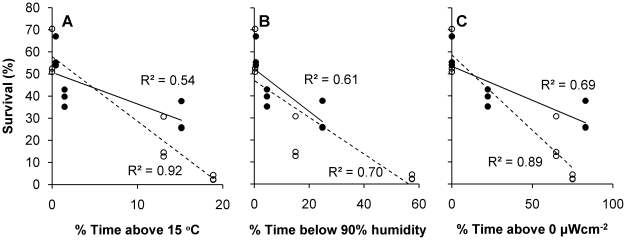
Relationship between *Galaxias maculatus* egg survival and environmental variables across all treatments. (A) time spent above 15°C, (B) time spent below 90% relative humidity and (C) time spent above 0 µW cm^−2^ UVB irradiance and survival of *Galaxias maculatus* eggs in Open (open circles) and Shaded (black circles) plots of three vegetation trimming treatments. Individual regression lines are fitted through the Open (dashed) and Shaded (solid) data points.

Egg survival was closely associated with the physical environment at the base of the riparian vegetation (Fig. 4), but there clearly was an interaction among physical variables that affected survival. Survival showed strong relationships with all physical variables, but the rate changed depending on whether the plots were shaded or not. Survival was lower in plots where the ground-level temperature exceeded 15°C frequently (F_1,14_ = 54.841, p<0.001), but this was modified by shading (F_1,14_ = 7.048, p<0.05). Plots where temperatures frequently exceeded 15°C, but which were Shaded, had better survival than Open plots (Fig. 4a). Egg survival was lower in plots where relative humidity fell below 90% frequently (F_1,14_ = 35.308, p<0.001; Fig. 4b), but this was modified by shading (F_1,14_ = 6.477, p<0.05). Relative humidity fell below 90% less frequently in the Shaded plots than it did in associated Open plots. For example, in the Cut Open plots, relative humidity was below 90% for 57% of the time and egg survival was very low (3.0±0.7%), but in the Cut Shaded plots relative humidity was below 90% less frequently (24.8%) and survival was greater (29.7±4.1%). Egg survival was lower in plots where UVB irradiance exceeded 0 µW cm^−2^ frequently (F_1,14_ = 51.259, p<0.001; Fig. 4c), but this was modified by shading (F_1,14_ = 8.722, p<0.05). Survival of eggs in plots exposed frequently to relatively high UVB levels was higher when they were Shaded than when they were Open.

**Figure 5:**
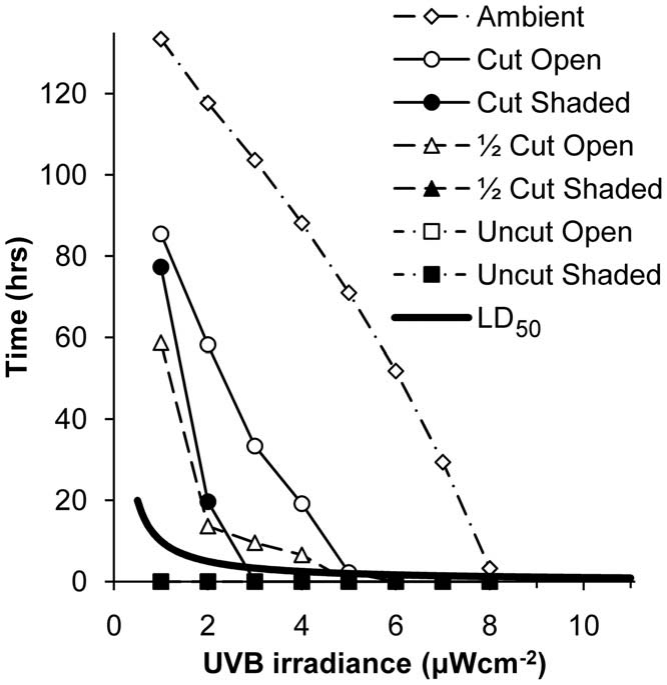
Total amount of time spent above levels of UVB irradiance in 18 plots during the two week experiment. For each treatment, and ambient conditions, the total amount of time (hrs) spent above each UVB irradiance level is shown. Also shown is the time required, at each UVB irradiance level, to reach the LD_50_ (3.6 kJm^−2^; Hickey C.W., unpublished results) for *Galaxias maculatus* eggs.

Ambient UVB irradiances exceeded 6 µW cm^−2^ for 52 of the 168 daylight hours during the two week experiment (Fig. 5). At ground-level, however, irradiances never surpassed 5.5 µW cm^−2^ even in the Cut Open plots. Nevertheless, accumulated energy levels in both Cut treatments (Open and Shaded), and the ½ Cut Open treatment were well in excess of the LD_50_ for *G. maculatus* eggs (3.6 kJm^−2^; Hickey C.W., unpublished results). Even at 1 µW cm^−2^, it takes less than 10 hrs exposure to UVB radiation to surpass this LD_50_ level, and the Cut Open treatment spent 85.5 hrs above this level.

**Figure 6:**
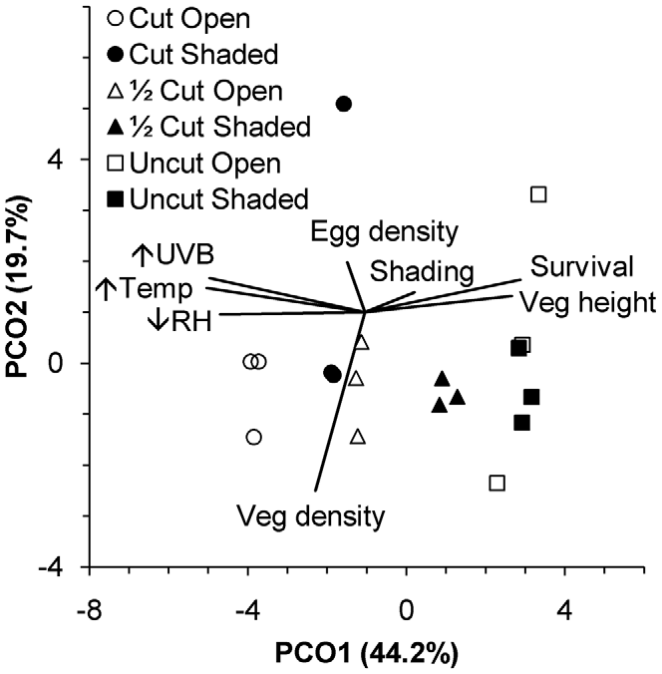
Principal coordinates biplot of *Galaxias maculatus* egg survival in 18 experimental plots with physical and biological predictor variables. Percentage of variation explained by individual axes is shown (↑UVB: time spent above 0 µW cm^−2^ UVB irradiance; ↑Temp: time spent above 15°C; ↓RH: time spent below 90% relative humidity).

In the multiple regression, the marginal tests showed that the amount of time a plot spent below 90% relative humidity, above 15°C and above 0 µW cm^−2^ UVB irradiance explained 77.0, 76.4 and 65.9% of the variation in *G. maculatus* egg survival, respectively (Table 1). Other variables individually explained a significant amount of variation in egg survival, but they were each correlated with variables that individually explained greater levels of variation. For example, Treatment explained 63.8% of the variation in egg survival, but it was correlated with relative humidity (r_16_ = 0.79, p<0.001), temperature (r_16_ = 0.88, p<0.001) and UVB radiation (r_16_ = 0.93, p<0.001). Since all of the potential driving variables are tightly correlated, it is difficult to tease out their relative effects on egg mortality. One way to do this is through an examination of interactions, which suggests how the effect of one parameter is altered by the value of another parameter. In the sequential test, highly correlated variables as well as those that did not explain significant amounts of variation were excluded (Table 1). Most of the variation in egg survival (83.2%) was explained by the combination of the time that plots spent below 90% relative humidity and the time the plots spent above 0 µW cm^−2^ UVB irradiance (Table 1). Including other variables in the model did not significantly increase the amount of variation that was explained (Table 1). Principal Coordinates analysis of egg survival in the experimental treatments illustrated the importance of the three physical variables (UVB irradiance, temperature and relative humidity), and the vegetation height that strongly influences these variables, in determining *G. maculatus* egg survival (Fig. 6).

**Table 1:** Tests for relationships between *Galaxias maculatus* egg survival in 18 experimental plots and biological and physical variables using non-parametric multivariate regression analysis (DISTLM).

Marginal tests				Sequential tests				
Variable	Pseudo-F	P	%Var	Variable	Pseudo-F	P	%Var	%Cum
↓RH	53.69	**0.0001**	77.0	↓RH	53.69	**0.0001**	77.0	77.0
↑Temp	51.83	**0.0001**	76.4	↑UV	5.45	**0.0343**	0.06	83.2
↑UV	30.86	**0.0002**	65.9	Shading	1.72	0.2100	0.02	85.0
Treatment	28.25	**0.0001**	63.8	↑Temp	1.67	0.2238	0.02	86.7
ΔVeg. density	26.50	**0.0001**	62.4	Veg. density	1.47	0.2499	0.01	88.2
Veg. height	19.46	**0.0007**	54.9	Veg. height	3.40	0.0980	0.02	91.0
ΔVeg. height	14.52	**0.0018**	47.6					
Shading	3.43	0.0842	17.7					
Veg. density	2.57	0.1227	13.9					
% *H. lanatus*	0.95	0.3445	0.05					
% *A. stolonifera*	0.43	0.5432	0.02					
% *S. phoenix*	0.22	0.6620	0.01					
Aspect	0.037	0.8511	0.002					
Egg density	0.007	0.9337	0.0005					

P-values<0.05 are in bold (%Var: the percentage of the variance in egg survival explained by that variable; %Cum: cumulative percentage of variance explained; ↓RH: time spent below 90% relative humidity; ↑Temp: time spent above 15°C; ↑UV: time spent above 0 µW cm^−2^ UVB irradiance; ΔVeg.: change in vegetation during experiment; % cover of three exotic pasture grasses).

## Discussion

Our results clearly show that the environment of the micro-site around *G. maculatus* eggs is of critical importance to survival, and that there is considerable buffering of the ambient conditions provided by the characteristics of the surrounding vegetation. The types of grasses favored by *G. maculatus* for egg-laying share the common characteristics of having open areas within their aerial root-mass suitable for egg deposition. *G. maculatus* swim among these on spring tides and lay eggs in dense clusters. This root-mass can be damaged when grasses are grazed by livestock or, in the case of urban stream-sides, by mowing. Previous work showed that fish often made poor choices because eggs had highly variable survival, depending on the height and density of vegetation in the particular sites in which they were laid [9]. We surmised that this was related to the characteristics of micro-sites and the tolerances of eggs to those conditions during the lunar month of their development. In this study, we have shown that temperature, relative humidity, and UVB radiation interact to affect egg survival, but that relative humidity and UV radiation have the strongest effects. We conclude that high levels of egg survival occur mostly in undisturbed vegetation, where relative humidity is above 90%, temperature below 15°C, and where UVB radiation is mostly filtered out.

Developing *G. maculatus* eggs have remarkable ability to withstand temperature fluctuations. The embryonic development sequence is very responsive to temperature changes without becoming abnormal [29]. For example, increasing ambient temperature from 8°C to 18°C reduces development times from 40 to 10 days [30]. Egg survival, however, is unaffected by temperatures ranging from 4°C to 22°C as long as relative humidity is consistently high [29], [31]. Temperature itself, therefore, is probably not lethal to eggs unless they are fully exposed to sustained temperatures exceeding 22°C [31]. It is more likely that reduced relative humidity associated with increasing temperature will have harmful effects on developing eggs.

*G. maculatus* eggs require high relative humidity for gaseous exchange to occur by diffusion across the moist chorion [10]. Therefore, sustained exposure to low humidity may prevent the eggs from respiring. Benzie [10] found that although *G. maculatus* eggs can recover from short-term dehydration, anything longer than 2–3 days was fatal. If *G. maculatus* eggs, like those of many other species of teleosts [32], [33], contain small glycogen stores, this short period may represent the extent of their anaerobic capacity. The micro-site climate beneath undisturbed riparian vegetation tends to dissociate humidity from temperature. Micro-sites can be relatively stable, with less extreme temperatures than the air above, and with a very high relative humidity and water vapor pressure [34]. The spawning habitat beneath undisturbed, dense riparian vegetation has been described as being “very close to an aquatic medium” [10].

In addition to buffering temperatures and humidity, vegetation around micro-sites shades developing *G. maculatus* eggs and significantly reduces UVB radiation reaching them. Many aquatic organisms, including fish [35], amphibians [36], [37] and intertidal invertebrates [38], [39], routinely deposit their egg masses in locations where the embryos have proven to be susceptible to periods of elevated UVB radiation. Frequently these are demersal (bottom-deposited) eggs with periodic emersion, but fully immersed fish eggs can also be vulnerable to UVB radiation, depending on the transparency and depth of water in which they are laid [19], [20], [40], [41]. Generally, immersed eggs are pelagic and buoyant, and drift near the surface with little protection from incident radiation. For diadromous populations of *G. maculatus*, the majority of egg development is terrestrial [9] but exceptions occur, such as in landlocked populations in Patagonia, where eggs are demersal and always immersed [42]. Depending on water transparency, natural UVB radiation causes a significant reduction in egg survival in these populations to a depth of c. 3 m [42]. For eggs developing terrestrially, we have shown here an equally high susceptibility to UVB radiation. In our experiment, any exposure to UVB radiation through trimming of riparian vegetation was associated with a significant decrease in the survival of *G. maculatus* eggs. Furthermore, it is noteworthy that this experiment was done in the latter half of the spawning season. Because of the annual cycle of UVB irradiances in New Zealand [43], the level of UVB radiation affecting eggs in the early part of the spawning season (February) would be almost 3 times those in this study (April). This increase in UVB radiation is highly likely to translate into even greater reductions in egg survival.

The exact nature of the relationship between temperature, relative humidity, UVB radiation, egg survival and vegetation type is still not entirely clear. In these experiments, done in natural surroundings, we could not experimentally control other physical factors, but instead measured them relative to vegetation and shading and their effects on egg survival. Laboratory-based experiments will be useful in delineating thresholds of these factors and the exact mechanisms that produce deleterious effects.

In this study, it was remarkable how such small variations to relatively small sites could have such a large effect on egg survival. These types of effects do not occur as rare patches in a natural landscape, but instead have become commonplace along the outlets of numerous streams and rivers, in some cases resulting in sink populations of adult *G. maculatus* with no effective place to lay eggs and have them develop [9]. It appears that associated changes in the physical conditions of egg laying sites are the major mechanism affecting good or poor egg survival. New Zealand has naturally high levels of UVB radiation, and the outlook for future levels is uncertain [43], [44]. It remains unknown whether the dominance of exotic pasture grasses, and the displacement of native vegetation, has exacerbated the penetration of UVB radiation to ground-level. In any case, the pathways of impacts associated with vegetational changes through human-induced disturbances are complex yet potentially devastating. This is perhaps especially so where a very small portion of habitat is required to complete the life history of a species, even a widely distributed one that traverses aquatic and marine environments.

## References

[1] Kennish MJ (2002). Environmental threats and environmental future of estuaries.. Environ Conserv.

[2] Nehlsen W, Williams JE, Lichatowich JA (1991). Pacific salmon at the crossroads: stocks at risk from California, Oregon, Idaho, and Washington.. Fisheries.

[3] McDowall RM (1968). *Galaxias maculatus* (Jenyns), the New Zealand Whitebait.. NZ Mar Dept Fish Res Bull.

[4] McDowall RM, Eldon GA (1980). The ecology of whitebait migrations (Galaxiidae: *Galaxias* spp.).. Fish Res Bull NZ Min Agr Fish.

[5] McDowall RM (1965). The composition of the New Zealand whitebait catch, 1964.. NZ J Sci.

[6] Rowe DK, Saxton BA, Stancliff AG (1992). Species composition of whitebait (Galaxiidae) fisheries in 12 Bay of Plenty rivers, New Zealand: evidence for river mouth selection by juvenile *Galaxias brevipinnis* (Günther).. NZ J Mar Freshw Res.

[7] McDowall RM (1990). New Zealand freshwater fishes: A natural history and guide..

[8] Taylor MJ (1996). How native fish spawn on land.. Water Atmos.

[9] Hickford MJH, Schiel DR (2011). Population sinks resulting from degraded habitats of an obligate life-history pathway.. Oecologia.

[10] Benzie V (1968). Some ecological aspects of the spawning behaviour and early development of the common whitebait *Galaxias maculatus attenuatus* (Jenyns).. Proc NZ Ecol Soc.

[11] Taylor MJ (2002). The national inanga spawning database: trends and implications for spawning site management.. Sci Cons.

[12] McDowall RM, Charteris SC (2006). The possible adaptive advantages of terrestrial egg deposition in some fluvial diadromous galaxiid fishes (Teleostei: Galaxiidae).. Fish Fish.

[13] McDowall RM, Mitchell CP, Brothers EB (1994). Age at migration from the sea of juvenile *Galaxias* in New Zealand (Pisces, Galaxiidae).. Bull Mar Sci.

[14] Baker CF (2004). Do invertebrates enjoy caviar too?. Water Atmos.

[15] Baker CF (2006). Predation of inanga (*Galaxias maculatus*) eggs by field mice (*Mus musculus*).. J Roy Soc NZ.

[16] Mitchell CP, Madgewick HH, Strickland RR, Van Boven RJ (1992). The use of larval fish as an aid to identifying whitebait spawning grounds, and the role of slugs as predators on whitebait eggs.. NZ Freshw Fish Misc Rep.

[17] Hickford MJH, Cagnon M, Schiel DR (2010). Predation, vegetation and habitat-specific survival of terrestrial eggs of a diadromous fish, *Galaxias maculatus* (Jenyns, 1842).. J Exp Mar Biol Ecol.

[18] Häder D-P, Kumar HD, Smith RC, Worrest RC (2007). Effects of solar UV radiation on aquatic ecosystems and interactions with climate change.. Photochem Photobiol Sci.

[19] Browman HI, Vetter RD, Rodriguez CA, Cullen JJ, Davis RF (2003). Ultraviolet (280-400 nm)-induced DNA damage in the eggs and larvae of *Calanus finmarchicus* G. (Copepoda) and Atlantic cod (*Gadus morhua*).. Photochem Photobiol.

[20] Olson MH, Colip MR, Gerlach JS, Mitchell DL (2006). Quantifying ultraviolet radiation mortality risk in bluegill larvae: Effects of nest location.. Ecol Appl.

[21] Wiegand MD, Young DLW, Gajda BM, Thuen DJM, Rittberg DAH (2004). Ultraviolet light-induced impairment of goldfish embryo development and evidence for photorepair mechanisms.. J Fish Biol.

[22] Yabu T, Ishibashi Y, Yamashita M (2003). Stress-induced apoptosis in larval embryos of Japanese flounder.. Fish Sci.

[23] McKenzie RL, Connor B, Bodeker GE (1999). Increased summertime UV radiation in New Zealand in response to ozone loss.. Science.

[24] McKenzie RL, Björn LO, Bais A, Ilyas M (2003). Changes in biologically active ultraviolet radiation reaching the Earth's surface.. Photochem Photobiol Sci.

[25] Underwood AJ (1981). Techniques of analysis of variance in experimental marine biology and ecology.. Oceanogr Mar Biol, Annu Rev.

[26] Anderson MJ (2002). DISTLM v.2: a FORTRAN computer program to calculate a distance-based multivariate analysis for a linear model..

[27] McArdle BH, Anderson MJ (2001). Fitting multivariate models to community data: a comment on distance-based redundancy.. Ecology.

[28] Anderson MJ, Gorley RN, Clarke KR (2008). PERMANOVA+ for PRIMER: guide to software and statistical methods..

[29] Benzie V (1968). Stages in the normal development of *Galaxias maculatus attenuatus* (Jenyns).. NZ J Mar Freshw Res.

[30] Mitchell CP (1989). Laboratory culture of *Galaxias maculatus* and potential applications.. NZ J Mar Freshw Res.

[31] Harzmeyer JR (2006). Effects of salinity and temperature on hatchability, and early development of *Galaxias maculatus* [Honours]: Deakin University.

[32] Finn RN (2007). The physiology and toxicology of salmonid eggs and larvae in relation to water quality criteria.. Aquat Toxicol.

[33] Terner C (1968). Studies of metabolism in embryonic development - III. Glycogenolysis and gluconeogenesis in trout embryos.. Comp Biochem Physiol.

[34] Geiger R, Aron RH, Todhunter P (2003). The climate near the ground..

[35] Hinrichs MA (1925). Modification of development on the basis of differential susceptibility to radiation I. *Fundulus heteroclitus* and ultraviolet radiation.. J Morphol.

[36] Marco A (2001). Effects of prolonged terrestrial stranding of aquatic *Ambystoma gracile* egg masses on embryonic development.. J Herpetol.

[37] Vredenburg VT, Romansic JM, Chan LM, Tunstall T (2010). Does UV-B radiation affect embryos of three high elevation amphibian species in California?. Copeia.

[38] Russell J, Phillips NE (2009). Synergistic effects of ultraviolet radiation and conditions at low tide on egg masses of limpets (*Benhamina obliquata* and *Siphonaria australis*) in New Zealand.. Mar Biol.

[39] Wraith J, Przeslawski R, Davis AR (2006). UV-induced mortality in encapsulated intertidal embryos: are mycosporine-like amino acids an effective sunscreen?. J Chem Ecol.

[40] Fukunishi Y, Masuda R, Yamashita Y (2010). Exposure of eggs to solar UV-B leads to reduced hatching rates in two sparid fishes, red sea bream *Pagrus major* and black sea bream *Acanthopagrus schlegeli*.. J Fish Biol.

[41] Huff DD, Grad G, Williamson CE (2004). Environmental constraints on spawning depth of yellow perch: the roles of low temperature and high solar ultraviolet radiation.. Trans Am Fish Soc.

[42] Battini M, Rocco V, Lozada M, Tartarotti B, Zagarese HE (2000). Effects of ultraviolet radiation on the eggs of landlocked *Galaxias maculatus* (Galaxiidae, Pisces) in northwestern Patagonia.. Freshw Biol.

[43] McKenzie RL, Liley JB, Gao W, Slusser JR, Schmoldt DL (2010). Balancing the risks and benefits of ultraviolet radiation.. UV radiation in global climate change: measurements, modelling and effects on ecosystems.

[44] Bais AF, Lubin D, Ennis CA (2007). Surface ultraviolet radiation: past, present and future.. Scientific assessment of ozone depletion: 2006.

